# Telemedicine use in Primary Care Associated with More Timely Access Without Unintended Subsequent Utilization for People with Dementia

**DOI:** 10.1007/s11606-024-09211-w

**Published:** 2024-11-18

**Authors:** Julia Adler-Milstein, Anjali Gopalan, Jie Huang, Christopher Toretsky, Mary Reed

**Affiliations:** 1 Division of Clinical Informatics and Digital Transformation, Department of Medicine, University of California San Francisco (UCSF), San Francisco, CA USA; 2 Division of Research, Kaiser Permanente Northern California (KPNC), Oakland, CA USA

## INTRODUCTION

Virtual and in-person care each has strengths and limitations,^1–3^ with sparse evidence to guide optimal selection. Evidence is particularly critical in the primary care setting for complex populations. One such population, people with dementia, benefit from avoiding travel to receive in-person care and may also receive more timely care.^4^ Yet, if telemedicine limitations make it challenging for primary care providers to deliver effective care to those with dementia (e.g., lack of physical exam coupled with cognitive challenges describing symptoms), telemedicine may result in higher post-visit utilization.^5^ In concerning cases, this could be an ED visit or lead to hospitalization. Subsequent in-person primary care would also signal inadequacy of telemedicine to initially address care needs but with less significant consequences. Drawing on evidence from two large health systems — one academic and one community, both of which had high primary care telemedicine use in the post-pandemic period for their dementia population^6^ — we assessed primary care timeliness and subsequent utilization, comparing telemedicine and in-person encounters.

## METHODS

Our sample included people with dementia with at least one primary care encounter in the post-COVID period (3/1/2021–2/28/2022) at UCSF (*N* = 419) and Kaiser Permanente Northern CA (KPNC; *N* = 18,037). Using EHR data from each organization, for each primary care encounter, we assessed the modality: in-person or telemedicine (video, phone). We also determined timeliness of the encounter: time in days between encounter scheduling and occurrence. Lastly, we assessed whether the individual had a hospitalization, ED encounter, in-person primary care visit, or none of these in the 14 days following the index encounter. Details on sample construction and measures are reported in prior work.^6^

By organization, we first assessed the relationship between encounter modality and timeliness, limiting the sample to the 0–30-day window to focus on those who needed near-term care. We used linear regression modeling that included nine covariates (Table 1) and also stratified by time since dementia diagnosis, number of comorbidities, and patient age to assess if benefits disproportionally accrued to sicker, more vulnerable individuals with dementia. Next, we assessed levels of near-term utilization following each encounter type using multinomial logistic regression models and the four-category dependent variable of 14-day subsequent care. We report adjusted rates and 95% confidence intervals. These were not restricted to the 0–30-day window and included the same covariates plus a categorical timeliness measure.

**Table 1:** People with Dementia Receiving Primary Care: Sample Statistics and Relationship between Encounter Modality and Timeliness

		**UCSF**			**KPNC**		
		**In Person**	**Telemedicine**		**In Person**	**Telemedicine**	
**Number of encounters**		619	502		17,535	21,541	
**Time between scheduling and encounter (timeliness)**							
0–3 days		136 (21.97%)	162 (32.27%)		8028 (45.78%)	14026 (65.11%)	
4–7 days		51 (8.24%)	56 (11.16%)		4158 (23.71%)	4882 (22.66%)	
8–13 days		62 (10.02%)	58 (11.55%)		3166 (18.06%)	1437 (6.67%)	
14–30 days		128 (20.68%)	103 (20.52%)		1939 (11.06%)	1050 (4.87%)	
> 30 days		242 (39.10%)	123 (24.50%)		244 (1.39%)	146 (0.68%)	
**Demographics**							
**Age**							
< 75		162 (26.17%)	114 (22.71%)		3275 (18.68%)	3783 (17.56%)	
75–79		108 (17.45%)	79 (15.74%)		3094 (17.64%)	3466 (16.09%)	
80–84		120 (19.39%)	83 (16.53%)		4085 (23.30%)	4769 (22.14%)	
85–89		127 (20.52%)	111 (22.11%)		4116 (23.47%)	5113 (23.74%)	
90 +		102 (16.48%)	115 (22.91%)		2965 (16.91%)	4410 (20.47%)	
**Sex**							
Female		378 (61.07%)	350 (69.72%)		10985 (62.65%)	14148 (65.68%)	
Male		241 (38.93%)	152 (30.28%)		6550 (37.35%)	7393 (34.32%)	
** Race/ethnicity**							
Asian		287 (46.37%)	201 (40.04%)		2416 (13.78%)	3150 (14.62%)	
Black or African American		60 (9.69%)	35 (6.97%)		1451 (8.27%)	2126 (9.87%)	
Latinx		49 (7.92%)	41 (8.17%)		2874 (16.39%)	3340 (15.51%)	
White		182 (29.40%)	203 (40.44%)		9823 (56.02%)	11724 (54.43%)	
Other/multi-race		41 (6.62%)	22 (4.38%)		969 (5.53%)	1199 (5.57%)	
Unknown/declined		0 (0.00%)	0 (0.00%)		2 (0.01%)	2 (0.01%)	
**Limited English proficiency**		225 (36.35%)	153 (30.48%)		2188 (12.48%)	2509 (11.65%)	
**Neighborhood SES quintile**							
1 — lowest socio-economic status		1 (0.16%)	0 (0.00%)		823 (4.69%)	797 (3.70%)	
2		25 (4.04%)	35 (6.97%)		2523 (14.39%)	3008 (13.96%)	
3		88 (14.22%)	64 (12.75%)		4411 (25.16%)	5253 (24.39%)	
4		166 (26.82%)	141 (28.09%)		5719 (32.61%)	7065 (32.80%)	
5 — highest socio-economic status		334 (53.96%)	259 (51.59%)		3867 (22.05%)	5190 (24.09%)	
Unknown		5 (0.81%)	3 (0.60%)		192 (1.09%)	228 (1.06%)	
**Patient portal access**		573 (92.57%)	479 (95.42%)		17336 (98.87%)	21048 (97.71%)	
**Distance from home to clinic**							
0– < 5 miles		484 (78.19%)	337 (67.13%)		9862 (56.24%)	11665 (54.15%)	
5 + miles		135 (21.81%)	165 (32.87%)		7667 (43.72%)	9860 (45.77%)	
Unknown		0 (0.00%)	0 (0.00%)		6 (0.03%)	16 (0.07%)	
**Time from dementia diagnosis**							
0 to < 1 years		62 (10.02%)	62 (12.35%)		2261 (12.89%)	2740 (12.72%)	
1 to < 2 years		117 (18.90%)	128 (25.50%)		4299 (24.52%)	4836 (22.45%)	
2 to < 3 years		148 (23.91%)	103 (20.52%)		4205 (23.98%)	5072 (23.55%)	
3 + years		292 (47.17%)	209 (41.63%)		6770 (38.61%)	8893 (41.28%)	
**Charlson Comorbidity Index**							
0		21 (3.39%)	11 (2.19%)		943 (5.38%)	1550 (7.20%)	
1		149 (24.07%)	131 (26.10%)		2156 (12.30%)	2225 (10.33%)	
2		122 (19.71%)	102 (20.32%)		3190 (18.19%)	3255 (15.11%)	
3		117 (18.90%)	72 (14.34%)		2739 (15.62%)	3143 (14.59%)	
4 +		210 (33.93%)	186 (37.05%)		8507 (48.51%)	11368 (52.77%)	
**Regression results: timeliness**		UCSF			KPNC		
Time between scheduling and encounter (days)	Stratum	Coeff	95% CI		Coeff	95% CI	
Overall		− 1.09	− 2.42	0.24	**− 2.26**	**− 2.37**	** − 2.14**
Time from dementia diagnosis (years)	0–3	− 1.32	− 3.01	0.37	** − 2.20**	**− 2.35**	** − 2.05**
	3 +	− 0.51	− 2.64	1.62	**− 2.38**	**− 2.60**	**− 2.16**
Charlson Comorbidity Index	0–4	**− 1.87**	**− 3.43**	**− 0.30**	**− 2.12**	**− 2.28**	**− 1.96**
	4 +	0.14	− 2.36	2.65	**− 2.52**	**− 2.72**	** − 2.32**
Patient age at encounter	< 90	− 1.06	− 2.51	0.39	**− 2.25**	**− 2.38**	**− 2.12**
	90 +	− 1.99	− 5.01	1.03	**− 2.29**	**− 2.51**	**− 2.07**

1. Standard errors adjusted for clustering of patients

2. Bolded results represent statistically significant relationships at p < 0.05

3. Models include the following covariates: age, sex, race/ethnicity, limited English proficiency, neighborhood SES, distance from home to clinic, years since dementia diagnosis, patient portal access (defined as the patient logging in to the portal within 1 year prior to the encounter date), and Charlson Comorbidity Index. Stratified models include all covariates except for the focal stratification variable

4. All models limited to encounters within 0–30 days after scheduling

## RESULTS

Table 1 presents sample statistics by encounter modality. UCSF had slightly more in-person encounters while KPNC had more telemedicine encounters. Assessing timeliness at KPNC, telemedicine was associated with timelier care overall and for all subgroups, with effect sizes of 2.12–2.52 days. At UCSF, for patients with fewer comorbidities, telemedicine was associated with 1.87 fewer days between scheduling and encounter than for patients with more comorbid conditions (95%CI: 0.30–3.43, Table 1).

**Figure 1:**
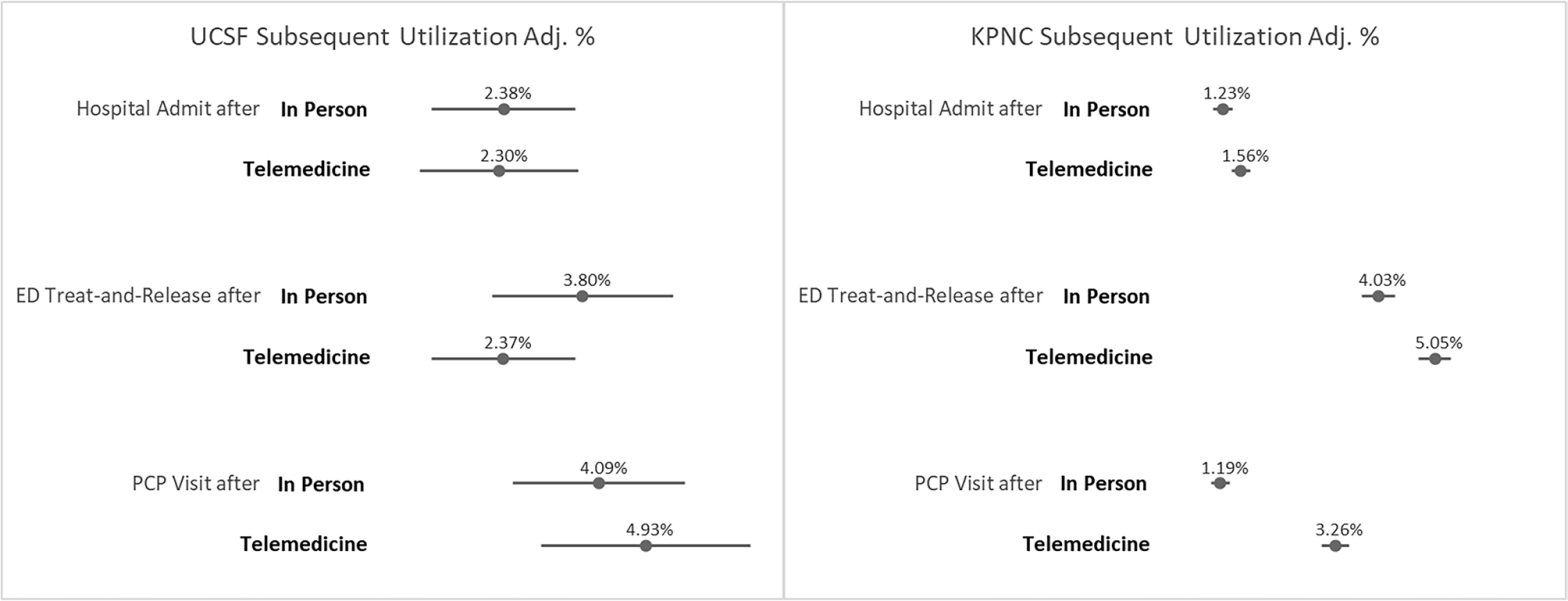
People with dementia receiving primary care: subsequent utilization (14 days after primary care encounter) by encounter modality (in person or telemedicine) at UCSF and KPNC. Notes: (1) Standard errors adjusted for clustering of patients; (2) Models include the following covariates: age, sex, race/ethnicity, limited English proficiency, neighborhood SES, distance from home to clinic, years since dementia diagnosis, patient portal access, Charlson Comorbidity Index, and number of days between booking and visit.

We found no evidence that telemedicine encounters had higher levels of subsequent utilization at UCSF (Fig. 1). At KPNC, hospitalizations did not differ by modality, but telemedicine encounters did have slightly higher levels of ED visits (5.05%, 95%CI: 4.75–5.34%) versus in-person visits (4.03%, 3.73–4.34%), and return in-person primary care visits within 14 days (3.26% after telemedicine encounter, 95%CI: 3.02–3.50% vs. 1.19% after in-person encounter, 95%CI: 1.02–1.36%).

## DISCUSSION

Telemedicine is enabling people with dementia to access primary care approximately 2 days earlier than in-person care, revealing that a known benefit of telemedicine extends to this population. While at KPNC those with more comorbidities experienced greater timeliness benefits, at UCSF, only the lower comorbidity group had more timely care. Underlying mechanisms should be assessed in future work but may be explained by both patient factors (e.g., at KPNC, sicker patients may be more adept at navigating telemedicine encounters) and provider factors (e.g., at UCSF, PCPs may feel less comfortable seeing sicker patients via telemedicine). Our work was also not able to assess the role of mobility status, functional status, or reason(s) for visit or subsequent utilization.

A small number of prior telemedicine studies found mixed impact on subsequent utilization (no change in ED visits, mixed impacts on hospitalizations).^5,7^ We found some evidence that telemedicine is associated with increased ED visits and in-person encounters. Given small magnitudes, our results suggest that telemedicine is largely being used appropriately to meet primary care needs of people with dementia. Taken together, we observe timeliness benefits without evidence of unintended consequences in the form of increased, near-term utilization.
